# Production of medium chain length polyhydroxyalkanoate in metabolic flux optimized *Pseudomonas putida*

**DOI:** 10.1186/1475-2859-13-88

**Published:** 2014-06-19

**Authors:** José Manuel Borrero-de Acuña, Agata Bielecka, Susanne Häussler, Max Schobert, Martina Jahn, Christoph Wittmann, Dieter Jahn, Ignacio Poblete-Castro

**Affiliations:** 1 Institute of Microbiology, Technische Universität Braunschweig D-38106, Braunschweig, Germany; 2 Department of Molecular Bacteriology, Helmholtz Centre for Infection Research, D-38124 Braunschweig, Germany; 3 Institute of Systems Biotechnology, Saarland University, D-66123 Saarbrücken, Germany; 4 Universidad Andrés Bello, Facultad de Ciencias Biológicas, Biosystems Engineering group, 8340176 Santiago, Chile; 5 Microbial Drugs group, Helmholtz Centre for Infection Research, D-38124 Braunschweig, Germany

**Keywords:** Medium chain length polyhydroxyalkanoate, *Pseudomonas putida*, Systems metabolic engineering, Pyruvate dehydrogenase, Glucose

## Abstract

**Background:**

*Pseudomnas putida* is a natural producer of medium chain length polyhydroxyalkanoates (mcl-PHA), a polymeric precursor of bioplastics. A two-fold increase of mcl-PHA production via inactivation of the glucose dehydrogenase gene *gcd*, limiting the metabolic flux towards side products like gluconate was achieved before. Here, we investigated the overproduction of enzymes catalyzing limiting steps of mcl-PHA precursor formation.

**Results:**

A genome-based *in silico* model for *P. putida* KT2440 metabolism was employed to identify potential genetic targets to be engineered for the improvement of mcl-PHA production using glucose as sole carbon source. Here, overproduction of pyruvate dehydrogenase subunit AcoA in the *P. putida* KT2440 wild type and the Δ*gcd* mutant strains led to an increase of PHA production. In controlled bioreactor batch fermentations PHA production was increased by 33% in the *acoA* overexpressing wild type and 121% in the *acoA* overexpressing Δ*gcd* strain in comparison to *P. putida* KT2440. Overexpression of *pgl-*encoding 6-phosphoglucolactonase did not influence PHA production. Transcriptome analyses of engineered PHA producing *P. putida* in comparison to its parental strains revealed the induction of genes encoding glucose 6-phosphate dehydrogenase and pyruvate dehydrogenase. In addition, NADPH seems to be quantitatively consumed for efficient PHA synthesis, since a direct relationship between low levels of NADPH and high concentrations of the biopolymer were observed. In contrast, intracellular levels of NADH were found increased in PHA producing organisms.

**Conclusion:**

Production of mcl-PHAs was enhanced in *P. putida* when grown on glucose via overproduction of a pyruvate dehydrogenase subunit (AcoA) in combination with a deletion of the glucose dehydrogenase (*gcd*) gene as predicted by *in silico* elementary flux mode analysis.

## Background

**Figure 1:**
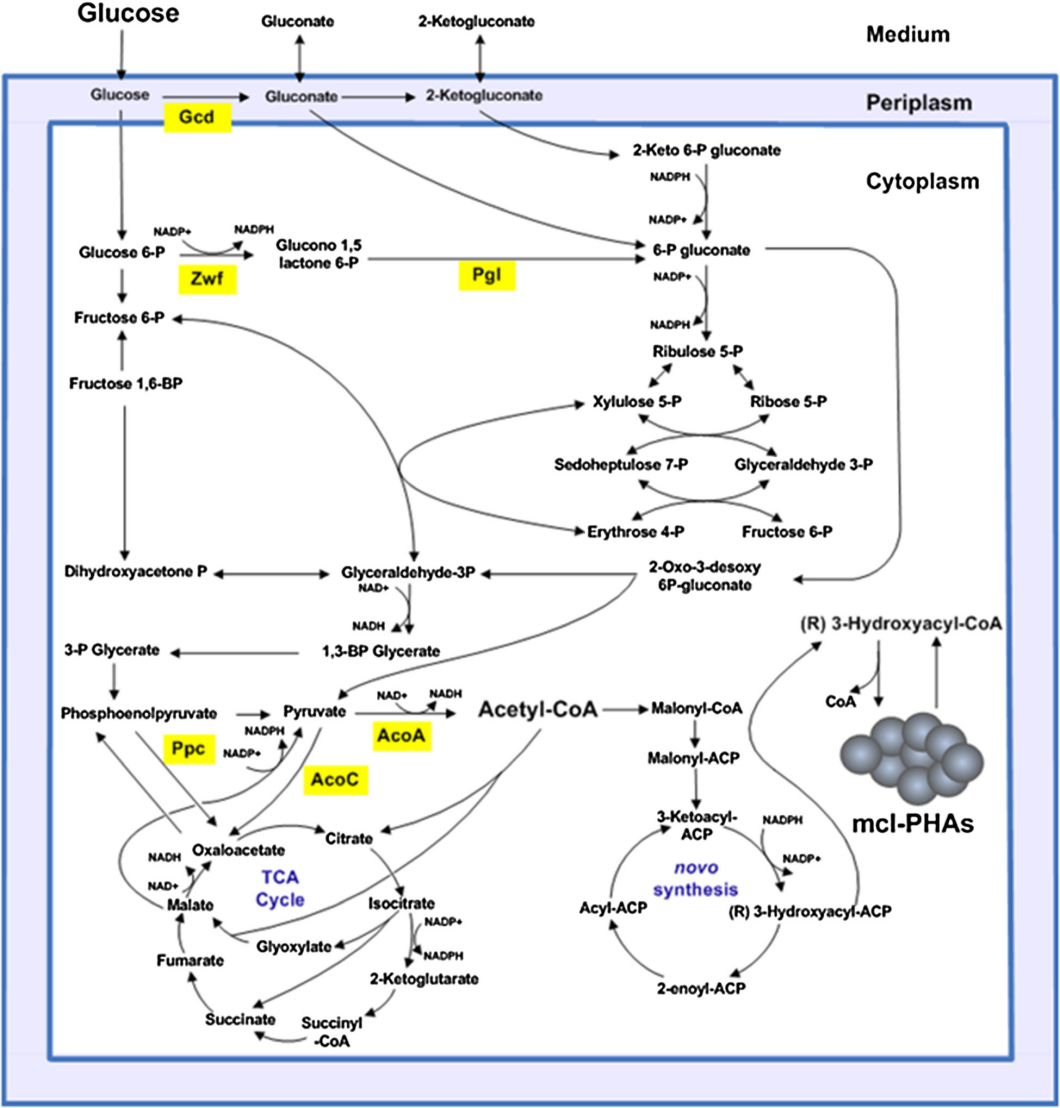
**Central metabolism of *P. putida *KT2440.** Enzymes of interest are indicated. Enzymatic steps with NAD^+^/NADP and NADP^+^/NADPH involvement are marked.

Metabolic engineering of microorganisms has significantly contributed to the biotechnological production of chemicals in a sustainable manner [1,2]. Insertion of heterologous pathways and modification of the host metabolism, via deletion or amplification of genes, has been a common procedure to enhance the synthesis of a desired compound [3,4]. Nevertheless, these modifications often evoke detrimental effects on the cell metabolism which lead to decreased growth and low performance of the constructed cell factory [2,3,5]. *In silico* reconstruction of the metabolism of bacterial production hosts in combination with metabolic flux modeling can help to design the optimal pathways towards a product of interest. Multiple efforts have been made to engineer microorganisms to obtain sustainable polymers which have the potential to replace petroleum-based plastics. In this regard, medium-chain-length polyhydroxyalkanoates (mcl-PHAs) are among the most promising biodegradable polymers synthesized by microorganisms which can be used in a wide range of applications [6]. Bacteria from the genus *Pseudomonas* are natural producers of mcl-PHAs since they have the entire machinery to synthesize these polyesters from different carbon sources (Figure 1) [7]. They accumulate these polymers as inclusion bodies in the cytoplasm promoted preferentially by a high carbon supply and the limitation of an inorganic nutrient such as nitrogen, oxygen, or phosphorous [8]. As other PHA-accumulating bacteria, *Pseudomonas putida* strains utilize PHAs as a reservoir of carbon and energy to cope with the changing environmental conditions of their natural habitats. For decades, studies for biotechnological mcl-PHA production focused on the use of fatty acid as carbon source [9-12]. This approach led to PHA accumulation in the cell, yielding more than 80% of its dry weight as PHA [13]. Additionally, engineering of the β-oxidation pathway in *P. putida* allowed for production of novel mcl-PHAs, which implies a broad range of applications in biomedicine and for biomaterials [10,14,15]. Much less attention has been given to the engineering of metabolic pathways involved in sugar or polyalcohol utilization in *P. putida*[16]. It was reported that inactivation of the isocitrate lyase gene resulted in a 1.7-fold increased production of mcl-PHA using gluconate as carbon source [17].

Different waste materials were tested as feedstock for PHA producing *Pseudomonas* species in order to reduce the overall production cost. Pseudomonas species accumulated mcl-PHAs when grown on sodium terephthalate produced from a PET pyrolysis, raw glycerol [18], polystyrene pyrolysis oil, and animal waste lipids [19]. Glucose is still one of the cheapest and most available feedstock for industrial polymer biosynthesis [20]. However, only small amounts of mcl-PHAs are currently synthesized utilizing glucose as carbon source [21,22]. Most metabolic approaches for the improvement of PHA production with glucose as carbon substrate were performed using *Escherichia coli*[23-25]. After cloning the PHA synthesis genes from *Cupriavidus necator* (formerly known as *Ralstonia eutropha*) into *E. coli* higher levels of NADPH versus NADP + were found essential to promote PHB synthesis. Consequently, bioengineering efforts on the rising of NADPH availability in turn to improve PHA synthesis on glucose were undertaken. Overproduction of glucose-6-phosphate dehydrogenase (G6PDH encoded by *zwf*) increased the intracellular NADPH levels and led to an increased accumulation of PHA by 41% [26]. A similar approach was carried out with *C. necator*, which resulted in an increased production of PHA in the presence of fructose [27]. It is known that *Pseudomonas* species prefer other carbon sources like organic acids and amino acids over glucose [28]. Consequently, our research focused on the development of *P. putida* strains which can accumulate high amounts of mcl-PHA on glucose as the sole carbon source. Recently, novel bioengineering approaches for an optimized production from glucose were undertaken [21]. Initially, a genome-based *in silico* model of the *P. putida* metabolism was established and used for the prediction of optimal fluxes towards PHA and its precursor molecules. Among a list of different genetic targets, elementary flux mode analysis suggested an inactivation of glucose dehydrogenase encoded by *gcd* to prevent undesired by-product formation and excretion. In agreement with the prediction from the *in silico* model the constructed *P. putida Δgcd* mutant strain showed an increased PHA production in batch [13] and Fed-Batch cultivations [29]. Here, we continued to experimentally verify the predictions of our *in silico* model for the optimization of mcl-PHA production in *P. putida*. We tested for the proposed positive impact of the overexpression of the genes for pyruvate dehydrogenase (*acoA*) and 6-phosphogluclactonase (*pgl*) on mcl-PHA production.

## Results and discussion

### Overexpression of the genes for the pyruvate dehydrogenase AcoA and 6-phosphoglucolactonase Pgl for improved PHA production

**Figure 2:**
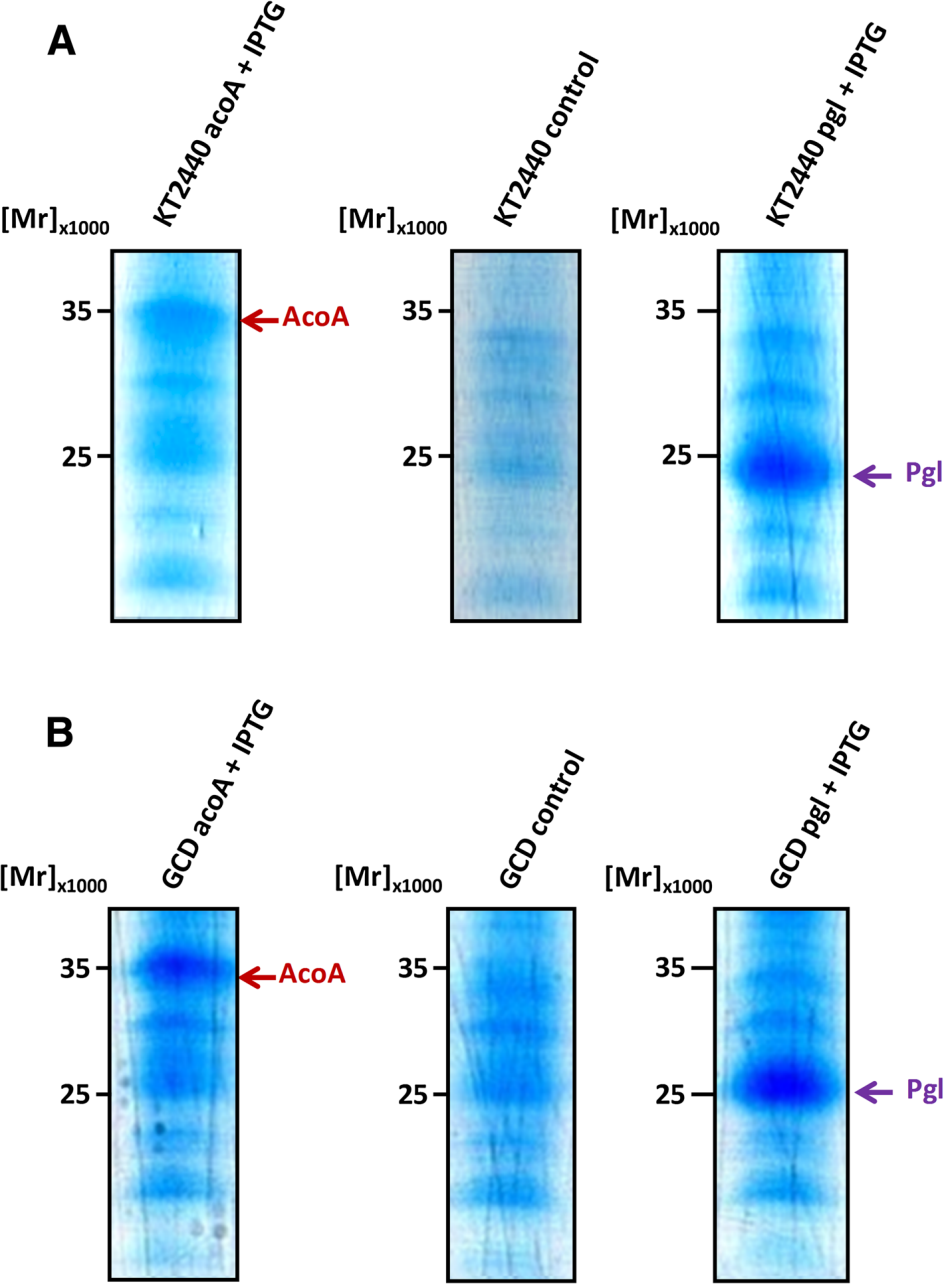
**SDS-PAGE gels demonstrating the recombinant production of pyruvate dehydrogenase and 6-phosphogluconolactonase by the recombinant *P. putida *strains. (A)** Shown the separations of whole cell protein extracts from KT2440 (lane 1), KT2440-*acoA* (lane 2), KT2440-*pgl* (lane 3), and **(B)** KT2440Δ*gcd* (lane 4), KT2440Δ*gcd-acoA* (lane 5) and KT2440Δ*gcd-pgl* (lane 6).

The *acoA* and *pgl* genes were separately cloned into the broad host range vector pSEVA424 and then transformed into the *P. putida* KT2440 and the Δ*gcd* mutant strains. A list of all used strains, vectors and primers are summarized in Table 1. Wild type *P. putida* KT2440 and the corresponding Δ*gcd* mutant strain carrying the vector without insert served as controls. Cells were grown in defined M9 mineral minimal medium supplemented with 20 (g/L) glucose and 62.5 μg/mL streptomycin as described in the Methods section. Protein production was induced at an initial OD_600nm_ of 0.05. Cells were harvested after 60 hours by centrifugation prior to protein composition analyses and PHA-extraction and quantification. SDS-PAGE analysis was used to assess the overproduction of the AcoA and the Pgl proteins. Successful overproduction of AcoA (M_r_ = 35,000) and Pgl (M_r_ = 23,000) was observed, which is in good agreement to the deduced relative molecular mass of approximately 34,700 Da for AcoA and 25,500 Da for Pgl (Figure 2).

**Table 1:** Bacterial strains, plasmids and primers used in this study

**Strains**	Relevant features/genotype*	**Source reference**
***E. coli***		
DH10b	F^-^*mcrA* Δ(*mrr*-*hsd*RMS-*mcr*BC) Φ80d*lac*ZΔM15 Δ*lac*X74*end*A1*rec*A1*deo*RΔ(*ara*,*leu*)7697*ara*D139 *gal*U*gal*K*nup*G*rps*L λ^-^	Invitrogen
XL10-Gold	*end*A1*gln*V44*rec*A1*thi*-1*gyr*A96*rel*A1*lac*HteΔ(*mcr*A)183 Δ(*mcr*CB-*hsd*SMR-*mrr*)173*tet*^R^ F’[*pro*AB*lacI*^q^ZΔM15 Tn*10*(Tet^R^ Amy Cm^R^)]	Stratagene
DH10b-*acoA*	DH10b carrying the *P. putida acoA* gene cloned in pJET1.2	This study
DH10b-*pgl*	DH10b carrying intermediate *pgl* gene cloned in pJET1.2	This study
XL10-Gold- *acoA*	XL10-Gold harboring *acoA* overexpression vector pSEVA*aco*	This study
XL10-Gold-*pgl*	XL10-Gold harboring *pgl* overexpression vector pSEVA*pgl*	This study
***P. putida***		
KT2440	Wild type	DSMZ
KT2440-*acoA*	KT2440 harboring *acoA* expression vector pSEVA424	This study
KT2440-*pgl*	KT2440 harboring *pgl* expression vector pSEVA424	This study
KT2440Δ*gcd*	KT2440 carrying *gcd* mutation	Poblete *et al.*, 2013[21]
KT2440Δ*gcd- acoA*	KT2440 carrying the *gcd* knockout mutation harboring *acoA* overexpression vector pSEVA*acoA*	This study
KT2440Δ*gcd-pgl*	KT2440 carrying the *gcd* knockout mutation harboring *pgl* overexpression vector pSEVA*pgl*	This study
**Plasmids**		
pJET1.2	Carrier vector, toxin expressed upon self-ligation, Ap^r^	Fermentas
pSEVA424	Expression vector for *P. putida*, IPTG-inducible, *tac* promoter, Sm^r^	De Lorenzo's Lab
pJET1.2-*acoA*	Plasmid pJET1.2 carrying *acoA* gene for propagation, Ap^r^	This study
pJET1.2-*pgl*	Plasmid pJET1.2 carrying *pgl* gene for propagation, Ap^r^	This study
pSEVA- *acoA*	*acoA* overexpression vector, based on pSEVA424, Sm^r^	This study
pSEVA-*pgl*	*pgl* overexpression vector, based on pSEVA424, Sm^r^	This study
**Primers**		
*acoA*Fw	5´CCG*GAATTC***AGGAGG** ***AAAAACAT*** ATGTCCAATCAACTCAGT3	This study
*acoA*Rv	5′TGC*TCTAGA*GCATCAGGGGTAGGCGACGTA3′	This study
*pgl*Fw	5´CCG*GAATTC***AGGAGG** ***AAAAACAT*** ATGGGAGGGCGTGGTATG3	This study
*pgl*Rv	5′TGC*TCTAGA*GCATCATGGGCACCAGTAGAT3′	This study

Ap^r^ = ampicillin resistance; Sm^r^ = streptomycin resistance.

^¥^Source the material was obtained from or reference. *EcoR*I and *Xba*I restriction sites are shown in italics. Shine Dalgarno (RBS) sequences are displayed in bold whereas the eight conserved nucleotides upstream the RBS are depicted in bold and italics.

### *Increased* mcl-PHA *production in* P. putida *by overexpression of the pyruvate dehydrogenase gene* acoA

**Figure 3:**
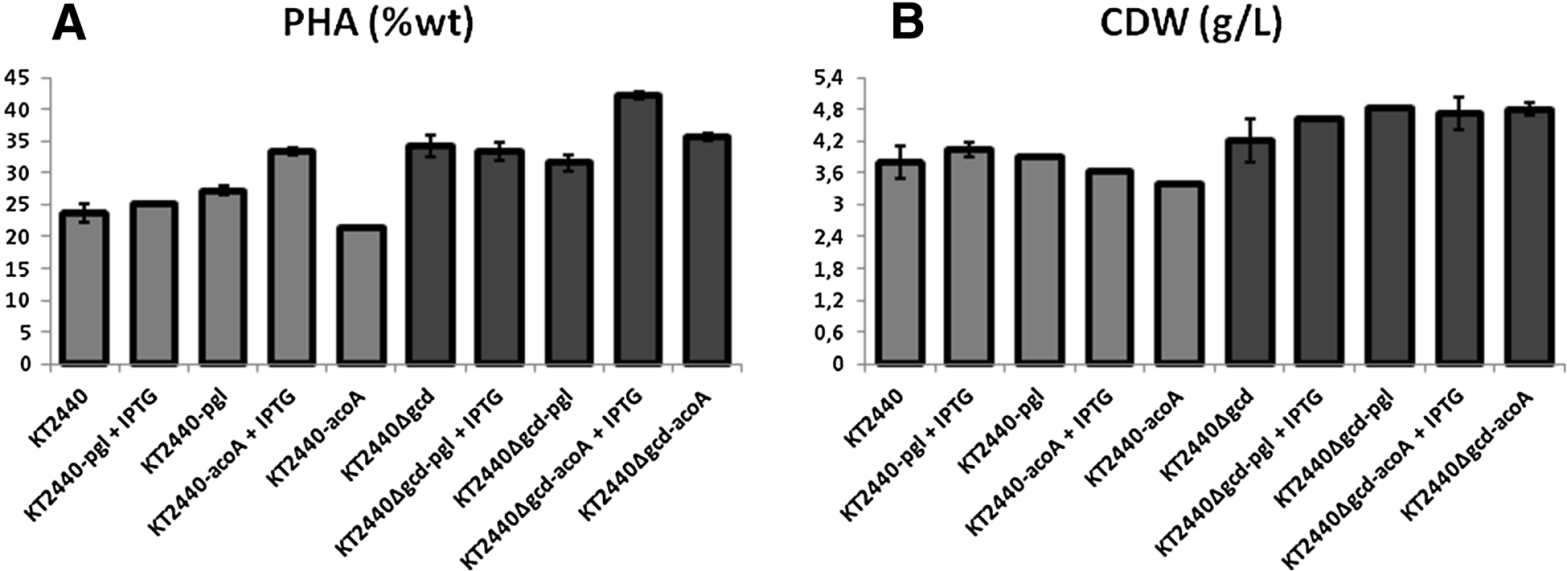
**mcl-PHA (A) content and cell dry weight (B) of different metabolically engineered indicated *P. putida *** **strains.**

In order to evaluate the effect of *acoA* and *pgl* overexpression on PHA versus biomass production GC-MS analyses of the extracted PHA and biomass determination were performed (Figure 3). Triplicate aerobic cultures of *P. putida* strains KT2440 (wt), KT2440-*acoA*, KT2440-*pgl*, KT2440Δ*gcd*, KT2440Δ*gcd*-*acoA*, and KT2440Δ*gcd*-*pgl* were tested. For this purpose, cells were grown in defined minimal medium for 60 hours until glucose as the sole carbon source was completely consumed (in all tested strains no glucose was detected via HPLC at 60 hours). As predicted by the *in silico* design the pyruvate dehydrogenase-producing KT2440-*acoA* and KT2440Δ*gcd*-*acoA* mutant strains both showed an increased PHA content amassing 33.3 wt % and 42.1 wt % as mcl-PHA, respectively (Figure 3A). This was not the case for the phosphoglucolactonase-overproducing KT2440-*pgl* (25.7 wt %) and KT2440Δ*gcd-pgl* (33.2 wt %) strains in which the PHA concentration remained at the basic level. In addition, no obvious major changes of the mcl-PHA momomer composition between the various producing *P. putida* strains were observed (Table 2). Next, the yield of biomass of engineered strains grown on glucose was determined. As initially desired, the biomass production of the various recombinant strains was found very similar to the one determined for the wild type *P. putida* KT2440 strain (Figure 3B).

**Table 2:** **Monomer composition of mcl-PHA produced by metabolically engineered *P. putida *** **strains in flask experiments**

**Strain**	**C6**^**∞**^	**C8**^**∞**^	**C10**^**∞**^	**C12**^**∞**^	**C12:1**^**∞**^	**C14**^**∞**^
**KT2440**	n.d.	11.8 ± 0.2	73.0 ± 0.1	4.7 ± 0.2	9.4 ± 0.7	0.8 ± 0.3
**KT2440Δ** ***gcd***	n.d.	14.0 ± 0.4	71.1 ± 0.8	4.6 ± 0.9	8.5 ± 0.4	1.1 ± 0.2
**KT2440-*acoA***	n.d.	13.0 ± 0.6	69.1 ± 1.2	3.4 ± 0.7	10.5 ± 0.2	3.9 ± 0.2
**KT2440Δ** ***gcd-acoA***	n.d.	14.8 ± 0.7	70.2 ± 1.4	5.1 ± 0.3	8.8 ± 0.1	0.7 ± 0.3
**KT2440-pgl**	n.d.	12.2 ± 0.9	74.1 ± 1.5	3.8 ± 0.5	9.9 ± 0.8	n.d.
**KT2440Δ** ***gcd-pgl***	n.d.	14.6 ± 0.3	73.1 ± 0.8	3.7 ± 0.2	8.1 ± 0.5	0.5 ± 0.1

n.d.: not detected, less than 0.2%.

^∞^: The data were determined by GC/MS and are given as relative molar fraction (%) of C6: 3-hydroxyexanoate, C8: 3-hydroxyoctanoate, C10:3-hydroxydecanoate, C12: 3-hydroxydodecanoate, C12:1: 3-hydroxy-5-cis-dodecanoate, and C14: 3-hydroxytetradecanoate.

In summary, the most efficient PHA producer was KT2440*Δgcd*-*acoA* strain, where the PHA concentration was doubled compared to *P. putida* wild type strain (Figure 3A) and enhanced by 21% compared to the KT2440Δ*gcd* mutant strain (Figure 3A). Thus, the predicted engineering strategy was successful in increasing the mcl-PHA synthesis of *P. putida*.

### *Batch fermentation in the bioreactors for controlled* mcl-PHA *production*

**Figure 4:**
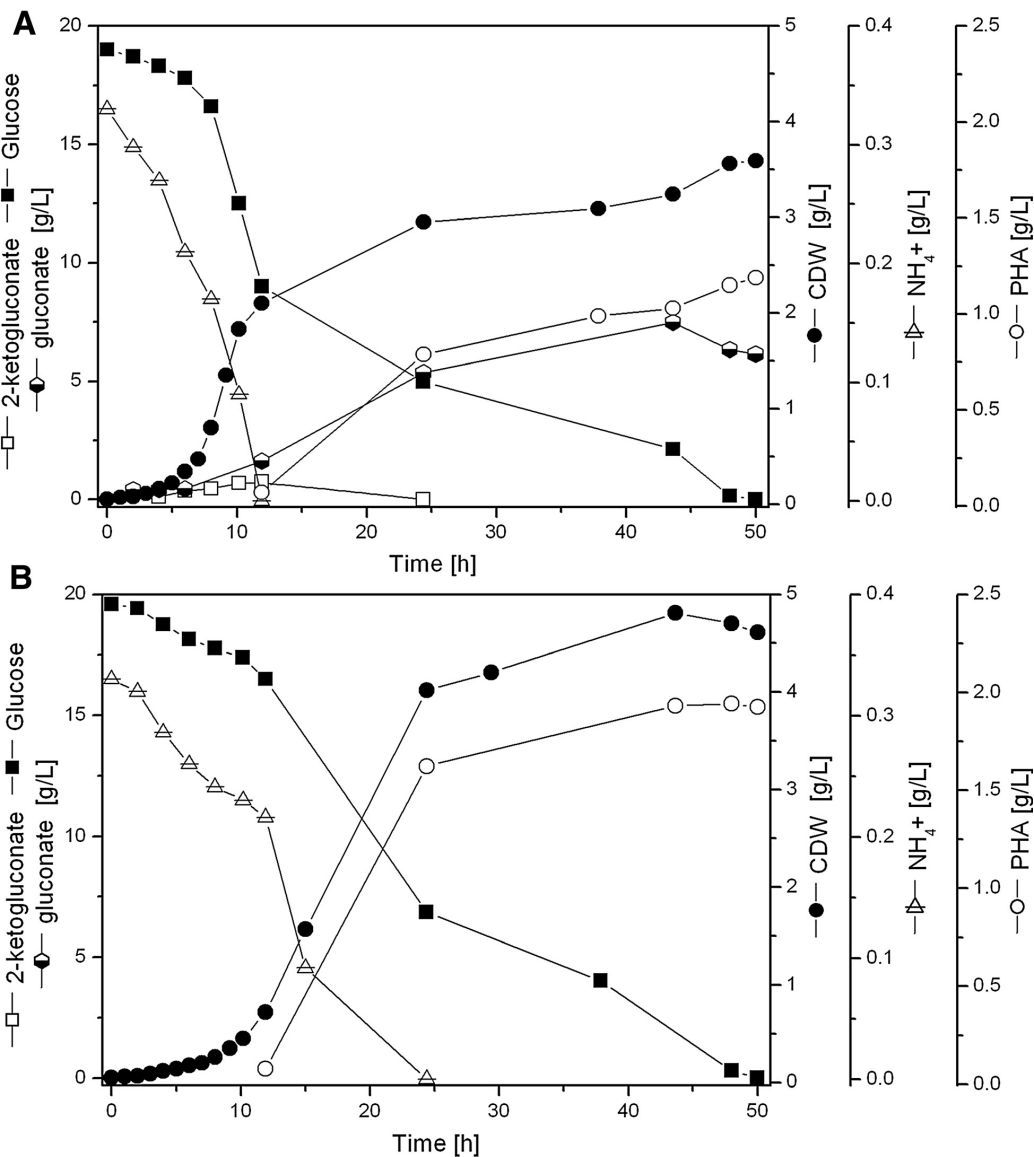
**Growth and mcl-PHA production of the metabolically engineered strains KT2440-*acoA *(A) and KT2440Δ *gcd- *acoA (B) in batch cultures.** Cells were grown in lab scale bioreactor in the presence of glucose 18.5 (g/L). Data are presented as mean values obtained from two independent experiments.

To ensure well-controlled mcl-PHA production using our metabolically engineered P*. putida* strains, aerobic batch fermentations of the KT2440-*acoA* and KT2440Δ*gcd*-*acoA* strains on glucose were performed in a bioreactor. Figure 4 shows the time-resolved measurements for mcl-PHA formation, biomass production and the corresponding concentrations of ammonium, gluconate, 2-ketogluconate, and glucose formation. As expected, the KT2440-*acoA* strain, gluconate and 2-ketogluconate were excreted into the medium during consumption of the available glucose (Figure 4A, Table 3). In contrast, during the fermentation of the flux optimized KT2440Δ*gcd*-*acoA* strain (Figure 4B) excreted organic acids were below the detection limit (Table 3). A recent study compared mcl-PHA synthesis in *P. putida* KT2440 and KT2442 when grown on gluconate [30]. *P. putida* KT2440 accumulated 17% of its CDW as PHA, whereas KT2442 only synthesized 1.7 wt % as PHA, demonstrating the large differences between the two *P. putida* strains. *P. putida* KT2440 can re-consume gluconate and 2-ketogluconate once secreted to the medium. We recently demonstrated that this process is not efficient to improve mcl-PHA synthesis [29], resulting in a lower volumetric PHA productivity as compared to the one shown by the Δ*gcd P. putida* mutant strain under the same growth conditions. Several investigations have shown that the volumetric productivity is the key parameter for the industrial production of PHAs and thus, any factor influencing this parameter affects the economics and cost competitiveness of PHAs synthesis via microbial fermentation [30-33]. Ammonium was completely consumed at 12 hours by the KT2440-*acoA* strain, while KT2440Δ*gcd*-*acoA* took more than 15 hours to deplete the entire amount of nitrogen. In addition, a large proportion of carbon was directed towards gluconate in the KT2440-*acoA* strain. More than 5 g/L of gluconate was produced after 24 h, (Figure 4A). Furthermore, the lower biomass of 2 g/L obtained for this strain prior ammonium limitation was most likely due to the increased production of cellular compounds including nucleic acids and proteins due to less PHA formation. These findings might explain the reduced PHA and biomass yield on glucose in comparison to the KT2440Δ*gcd*-*acoA* mutant strain. The genetic modification of the KT2440Δ*gcd*-*acoA* led to the lowest μ_max_ of all strains (0.25 h^-1^ versus 0.56 h^-1^ for KT2440). Nevertheless, the KT2440Δ*gcd*-*acoA* strain biomass (CDW = 4.71 g/L after 50 hrs) and mcl-PHA 42.1 wt % contents were higher in comparison to the KT2440-*acoA* strain (CDW = 3.63 g/L after 50 hrs and mcl-PHA = 33.3 wt %). To our knowledge this is the highest mcl-PHA accumulation ever reported using a *P. putida* strain in the presence of glucose in batch cultures.

**Table 3:** **Physiological parameters of the wild type *P. putida *** **KT2440 and metabolically engineered strains in batch culture grown with of glucose**

**Strain**	***μ***_**max **_**(h**^**-1**^**)**	**Y**_**X/S**_^**§ **^**(g/g)**	**Y**_**2-ketoglut/S **_**(g/g)**	**Y**_**gluconate/S **_**(g/g)**	**Y **_**PHA/S **_**(g/g)**
**KT2440**	0.56	0.22	<0.001	0.64	0.05
**KT2440Δ** ***gcd***	0.42	0.23	<0.001	<0.001	0.08
**KT2440-*acoA***	0.40	0.21	0.04	0.38	0.07
**KT2440Δ** ***gcd-acoA***	0.25	0.25	<0.001	<0.001	0.11
**KT2440** ***-pgl***	0.33	0.21	0.07	0.55	0.05
**KT2440Δ** ***gcd-pgl***	0.21	0.24	<0.001	<0.001	0.08

^§^ Y_x/s_ is calculated based on the total biomass concentration (true biomass + PHA content). Standard deviations of less than 10% were obtained in 3 independent experiments.

### *Transcriptome analysis of the engineered* P. putida *PHA production strains*

Glucose is metabolized in *P. putida* that utilizes three pathways converging at the level of 6-phosphogluconate prior to the formation of 2-keto-3-deoxy-6-phosphogluconate (KDPG; [34]). The repressor HexR regulates the expression of major genes of the pathway including the *zwf-1*, *pgl*, *eda* operon and the *gap1* gene encoding glyceraldehyde-3-phosphate dehydrogenase. Accumulation and binding of KDPG to HexR lead to derepression [35-37]. Furthermore, the genes of PHA synthesis are subjected to a complex control by catabolite regulation and various stress responses mediated by the complex RNA-dependent GacS and Crc regulating systems, besides others [38-40]. In order to understand the gene regulatory adaptation of the newly engineered strains to enhanced PHA production the gene expression compared to the parental strains was analyzed using next generation sequencing of transcripts (RNA_Seq_). We are fully aware that major adaptation processes might take place at the level of enzyme regulation which cannot be made visible with the employed approach. Samples were taken after 60 hours of cultivation, when the PHA concentration in the cell reached its highest level. Strict cut-off parameters (log change > 2 and a *p*-value < 0.05) were applied. First, the gene expression of *acoA* overexpressing strains was compared to the wild type KT2440 strain and the KT2440Δ*gcd* mutant strain (Table 2). Overall, 53 open reading frames (ORFs) were found differentially transcribed (12 up-regulated and 41 down-regulated) between the KT2440 wild type strain with or without overexpressed *acoA*. Moreover, 108 ORFs (59 up-regulated and 49 down-regulated) were found changed in their transcriptome between the KT2440Δ*gcd* strain with or without overexpressed *acoA*. As expected, the strongest up-regulated gene in both variant strains overexpressing *acoA* was PP_0555 (*acoA*). Interestingly, also PP_0554 (*acoB*) was found upregulated, however to a lesser extent (Table 4). Most of the genes encoding for enzymes related to the central and PHA synthesis metabolic pathways showed only slight variations in their expression levels (Table 4). Interestingly, the increased flux toward acetyl-CoA and as a consequence citrate induced the expression of the isocitrate lyase gene, which encodes the first enzyme of the glyoxylate shunt. One metabolic consequence is a reduced NADH production in the TCA cycle. A second consequence might be the channeling of acetyl-CoA into the glyoxylate cycle. In agreement, it was previously shown that inactivation of the isocitrate lyase gene leads to increased PHA production [17]. Overall, the gene expression data do not provide conclusive explanations for the observed increase in PHA production. Most likely regulatory effects at the enzyme level might be responsible.

**Table 4:** **Expression profile of the genes belonging to PHA biosynthesis and central metabolic pathways in the metabolically engineered *P. putida *** **strains compared to their parental strains**

**Gene name**	**Locus tag**	**Description**	**Fold change**^**§**^	
			**Δ** ***gcd-acoA***	**KT2440-acoA**
**PHA synthesis**				
*phaI*	PP5008	PHA granule-associated	0.7	0.9
*phaF*	PP5007	PHA granule-associated	-0.3	0.2
*phaC1*	PP5003	PHA polymerase	-0.2	0.7
*phaC2*	PP5005	PHA polymerase	-0.6	-0.2
*phaZ*	PP5004	PHA depolymerase	0.3	0.5
*phaD*	PP5006	Transcriptional regulator	-0.4	0.3
*phaG*	PP1408	Acyl-transferase	**1.5**	-0.1
**Transporters**				
*oprB-1*	PP1019	Porin	-0.7	**-1.0**
*oprB-2*	PP1445	Porin	-0.5	-0.6
**Glycolysis/gluconeogenesis**				
*glk*	PP1011	Glucokinase	0.2	-0.2
*pgi*	PP1808	Glucose-6-phosphate isomerase	-0.5	0.0
*fbp*	PP5040	Fructose-1,6-bisphosphatase	0.6	0.4
*fda*	PP4960	Fructose-1,6-bisphosphate aldolase	0.9	0.1
*tpiA*	PP4715	Triosephosphate isomerase	0.8	0.6
*gap1*	PP1009	GAP dehydrogenase, type I	**-1.2**	-0.4
*gap2*	PP2149	GAP dehydrogenase, type II	0.2	0.3
*pgk*	PP4963	Phosphoglycerate kinase	0.2	0.1
*pgm*	PP5056	Phosphoglyceromutase	0.4	0.1
*eno*	PP1612	Phosphopyruvate hydratase	0.3	0.0
*pyk*	PP1362	Pyruvate kinase	-0.3	0.4
**Pentose phosphate pathways**				
*zwf1*	PP1022	G6P dehydrogenase	0.9	0.2
*zwf2*	PP4042		0.1	-0.3
*zwf3*	PP5351		0.2	0.4
*pgl*	PP1023	6-phosphogluconate dehydrogenase	0.5	-0.9
*gnd*	PP4043	6-phosphoglucolacto dehydrogenase	-0.6	-0.1
*gnuK*	PP3416	Carbohydrate kinase	**-1.1**	-0.6
*kguK*	PP3378	Dehydroglucokinase	0.45	-0.4
*kguD*	PP3376	2-Ketogluconate 6-phosphate reductase	-0.7	-0.5
*rpiA*	PP5150	Ribose-5-phosphate isomerase A	0.3	0.0
*rpe*	PP0415	Ribulose-phosphate 3-epimerase	-0.5	-0.1
*tktA*	PP4965	Transketolase	0.3	0.7
*tal*	PP2168	Transaldolase B	0.4	0.3
**Entner-Doudoroff pathway**				
*edd*	PP1010	6-Phosphogluconate dehydratase	-0.3	-0.3
*eda*	PP1024	KDPG aldolase	0.0	-0.5
**Pyruvate metabolism**				
*acoA*	PP0555	Pyruvate dehydrogenase	**12.4**	**5.3**
*acoB*	PP0554	Pyruvate dehydrogenase	**1.5**	**1.0**
*acoC*	PP0553	Pyruvate dehydrogenase	0.7	0.7
	PP0545	Aldehyde dehydrogenase	0.1	**-1.0**
*acsA*	PP4487	Acetyl-CoA synthetase	0.8	**1.4**
*accC-2*	PP5347	Pyruvate carboxylase	0.5	0.2
*ppsA*	PP2082	Phosphoenolpyruvate synthase	0.5	0.4
*ppc*	PP1505	Phosphoenolpyruvate carboxylase	0.5	0.4
**TCA cycle**				
*gltA*	PP4194	Citrate synthase	0.7	0.7
*acnA*	PP2112	Aconitate hydratase	0.4	-0.4
*acnB*	PP2339	Aconitate hydratase	0.7	0.3
*icd*	PP4011	Isocitrate dehydrogenase	0.3	-0.5
*sucA*	PP4189	2-Oxoglutarate dehydrogenase	-0.7	-0.2
*sucD*	PP4185	Succinyl-CoA synthetase sub alpha	-0.1	-0.3
*sucC*	PP4186	Succinyl-CoA synthetase sub beta	0.2	0.3
*sdhA*	PP4191	Succinate dehydrogenase	0.3	0.4
*fumC*	PP0944	Fumarate hydratase	0.5	**-1.1**
*mdh*	PP0654	Malate dehydrogenase	-0.4	**-1.3**
**Glyoxylate shunt**				
*aceA*	PP4116	Isocitrate lyase	**2.9**	**1.3**
*glcB*	PP0356	Malate synthase	0.8	0.8

^§^Bold numbers represent a differentiated gene expression pattern.

Next, the *pgl* overexpressing strains without increased PHA production were analyzed for potential compensatory gene regulatory activities. Of the 86 ORFs differentially expressed in KT2440-*pgl* mutant strain compared to the wild type strain, 77 corresponded to genes that were transcriptionally up-regulated, whereas 9 genes were found with decreased expression levels (Table 5). For KT2440Δ*gcd*-*pgl* mutant strain compared to the KT2440Δ*gcd* mutant, 15 genes were found down-regulated while 117 ORFs were found induced. As expected, the *pgl* overexpressing strains revealed an increase of *pgl* mRNA. Glucose 6-phosphate dehydrogenase (encoded by PP_1022, *zwf*) was found to be highly up-regulated (>12 fold) in both strains harboring the overexpression system (Table 5). Obviously, the initial goal to enhance the flux from glucose toward KDPG was achieved. The increased amounts of the metabolite might have enhanced *zwf-1* expression via HexR derepression. Furthermore, expression differences were detected for the pyruvate metabolism, where the genes encoding pyruvate dehydrogenase (PP_0554), pyruvate carboxylase (PP_5347) and phosphoenoylpyruvate (PP_1505) showed differential expression levels (Table 5). At least for the wild type strain harboring the overexpressed *pgl* a problematic rearrangement of pyruvate metabolization occurred which might be responsible for the observed production phenotype. While the pyruvate dehydrogenase gene *acoA* was found reduced, the pyruvate carboxylase gene *acoC-2* was found induced (Table 5). In addition, induction of the phosphoenolpyruvate carboxylase gene *ppc* was detected. The KT2440Δ*gcd* mutant carrying overexpressed *pgl* did not reveal this response. For the *pgl* overexpression the observed gene regulatory response provides reasonable explanations for the observed PHA production phenotype.

**Table 5:** Expression profile of the genes belonging to PHA biosynthesis and central metabolic pathways in the metabolically engineered strains compared to their parental strains

**Gene name**	**Locus tag**	**Description**	**Fold change**^**§**^	
			**Δ** ***gcd-pgl***	**KT2440** ***-pgl***
**PHA synthesis**				
*phaI*	PP5008	PHA granule-associated	0.6	-0.3
*phaF*	PP5007	PHA granule-associated	0.3	-0.4
*phaC1*	PP5003	PHA polymerase	-0.8	-0.1
*phaC2*	PP5005	PHA polymerase	-0.7	-0.4
*phaZ*	PP5004	PHA depolymerase	-0.4	0.0
*phaD*	PP5006	Transcriptional regulator	0.2	0.1
*phaG*	PP1408	Acyl-transferase	**2.1**	0.2
**Transporters**				
*oprB-1*	PP1019	Porin	0.4	-0.1
*oprB-2*	PP1445	Porin	-0.4	-0.4
**Glycolysis/gluconeogenesis**				
*glk*	PP1011	Glucokinase	0.3	0.2
*pgi*	PP1808	Glucose-6-phosphate isomerase	-0.4	0.0
*fbp*	PP5040	Fructose-1,6-bisphosphatase	0.3	-0.1
*fda*	PP4960	Fructose-1,6-bisphosphate aldolase	0.6	-0.1
*tpiA*	PP4715	Triosephosphate isomerase	0.4	0.5
*gap1*	PP1009	GAP dehydrogenase, type I	-0.4	-0.2
*gap2*	PP2149	GAP dehydrogenase, type II	-0.3	-0.2
*pgk*	PP4963	Phosphoglycerate kinase	0.1	0.0
*pgm*	PP5056	Phosphoglyceromutase	0.5	-0.3
*eno*	PP1612	Phosphopyruvate hydratase	0.5	0.1
*pyk*	PP1362	Pyruvate kinase	-0.5	0.6
**Pentose phosphate pathways**				
*zwf1*	PP1022	G6P dehydrogenase	**5.2**	**3.6**
*zwf2*	PP4042		0.2	-0.6
*zwf3*	PP5351		-0.1	-0.3
*pgl*	PP1023	6-phosphogluconate dehydrogenase	**9.3**	**7.8**
*gnd*	PP4043	6-phosphoglucolacto dehydrogenase	0.2	-0.3
*gnuK*	PP3416	Carbohydrate kinase	-0.1	0.3
*kguK*	PP3378	Dehydroglucokinase	0.6	0.5
*kguD*	PP3376	2-Ketogluconate 6-phosphate reductase	0.1	-0.1
*rpiA*	PP5150	Ribose-5-phosphate isomerase A	0.3	-0.4
*rpe*	PP0415	Ribulose-phosphate 3-epimerase	0.2	-0.1
*tktA*	PP4965	Transketolase	0.7	0.3
*tal*	PP2168	Transaldolase B	0.2	0.1
**Entner-Doudoroff pathway**				
*edd*	PP1010	6-Phosphogluconate dehydratase	0.2	0.1
*eda*	PP1024	KDPG aldolase	0.4	-0.2
**Pyruvate metabolism**				
*acoA*	PP0555	Pyruvate dehydrogenase	**1.3**	**-2.7**
*acoB*	PP0554	Pyruvate dehydrogenase	0.7	-0.8
*acoC*	PP0553	Pyruvate dehydrogenase	0.5	**-1.2**
	PP0545	Aldehyde dehydrogenase	0.5	-0.9
*acsA*	PP4487	Acetyl-CoA synthetase	-0.3	-0.6
*accC-2*	PP5347	Pyruvate carboxylase	0.8	**2.3**
*ppsA*	PP2082	Phosphoenolpyruvate synthase	0.2	-0.1
*ppc*	PP1505	Phosphoenolpyruvate carboxylase	0.0	**1.4**
**TCA cycle**				
*gltA*	PP4194	Citrate synthase	-0.2	0.7
*acnA*	PP2112	Aconitate hydratase	0.8	-0.5
*acnB*	PP2339	Aconitate hydratase	-0.2	0.3
*icd*	PP4011	Isocitrate dehydrogenase	-0.3	-0.3
*sucA*	PP4189	2-Oxoglutarate dehydrogenase	-0.6	0.2
*sucD*	PP4185	Succinyl-CoA synthetase sub alpha	-0.2	0.5
*sucC*	PP4186	Succinyl-CoA synthetase sub beta	-0.1	0.1
*sdhA*	PP4191	Succinate dehydrogenase	-0.7	0.3
*fumC*	PP0944	Fumarate hydratase	**1.1**	-0.4
*mdh*	PP0654	Malate dehydrogenase	0.8	-0.8
**Glyoxylate shunt**				
*aceA*	PP4116	Isocitrate lyase	**1.2**	**-1.1**
*glcB*	PP0356	Malate synthase	-0.4	-0.1

*p*-value < 0.05.

^§^Bold numbers represent a differentiated gene expression pattern.

### The role of NADPH and NADH in P. putida PHA production

The production of mcl-PHAs in *P. putida* KT2440 requires NADPH + H^+^. *P. putida* catabolizes glucose via the Entner-Doudoroff pathway and hence the NADPH + H^+^ producing glucose-6-phosphate dehydrogenase (Zwf) is highly active. However, the attempts to further increase the metabolic flux through this step by overproducing the rate-limiting, succeeding enzyme (Pgl) failed to increase PHA production (Figure 1). However, the flux of carbon towards PHA was most likely hampered at the pyruvate metabolization step in the *pgl* overexpression strain. Interestingly, the transcriptome analyses revealed an increased *zwf* gene expression. In order to determine the contribution of the NADPH + H^+^ level in *P. putida* to PHA production, NADPH + H^+^ versus NADP^+^ levels were determined for the various bioengineered strains (Table 6). The ratios of NADPH to NADP^+^ were measured using UV/Vis spectrometry and quantified according to their colorimetric changes at λ = 450 nm. At the maximum mcl-PHA accumulation in the cell (60 h culture), we found the NADPH/NADP^+^ ratios of all the PHA-overproducing strains were lower compared to the corresponding wild type *P. putida* KT2440 (Table 6). Clearly, the employed metabolic engineering strategy increased the polyester production in both strains (Table 3, Figure 3). As a consequence of this increased flux of carbon into PHA synthesis, more NADPH is needed to turn 3-ketoacyl-ACP into (R)-3-hydroxyacyl-ACP (Figure 1). Therefore, the levels of NADPH/NADP^+^ were lower for the engineered *P. putida* strains (Table 6), in which pyruvate dehydrogenase was overproduced. A totally different picture was observed for the strains in which the *pgl* gene was overexpressed. Here, an increase of the NADPH/NADP^+^ levels was detected (Table 6). As already deduced from the transcriptome data, the initial flux through the Entner-Doudoroff pathway might have been induced, leading to a KDPG accumulation and an increased *zwf-1* expression. Consequently, sufficient NADPH was produced. However, the increased flux of carbon towards PHA was lost at least in one case at the *pgl* overexpression strains. Overall, gene and most likely enzyme activity regulatory phenomena prohibited further flux towards PHA production and resulted in increased NADPH + H^+^ levels.

**Table 6:** **Levels of the NAD**^**+**^**, NADH, NADP**^**+**^**, and NADPH cofactors and their ratios of various *P. putida *** **strains under PHA producing conditions**

**Strain**		Cofactor level (mmol/g rCDW)*			**Cofactor ratio**	
	**NAD**^**+**^	**NADH**	**NADP**^**+**^	**NADPH**	**NADH/NAD**^**+**^	**NADPH/NADP**^**+**^
KT2440	**7.62 ± 0.86**	**0.67 ± 0.08**	**3.22 ± 0.38**	**2.67 ± 0.07**	**0.09**	**0.83**
KT2440Δ*gcd*	**6.01 ± 0.67**	**1.75 ± 0.36**	**4.49 ± 0.12**	**1.31 ± 0.32**	**0.30**	**0.29**
KT2440-*acoA*	**6.25 ± 0.42**	**2.02 ± 0.15**	**6.48 ± 0.48**	**0.87 ± 0.27**	**0.32**	**0.13**
KT2440Δ*gcd-acoA*	**6.88 ± 0.33**	**2.94 ± 0.15**	**3.98 ± 0.46**	**0.96 ± 0.36**	**0.43**	**0.24**
KT2440-*pgl*	**5.59 ± 0.14**	**1.82 ± 0.10**	**2.58 ± 0.40**	**3.07 ± 0.41**	**0.33**	**1.19**
KT2440Δ*gcd-pgl*	**10.81 ± 0.50**	**2.18 ± 0.38**	**2.30 ± 0.15**	**3.71 ± 1.23**	**0.20**	**1.62**

*rCDW: residual cell dry weight.

By overproducing the AcoA subunit of the pyruvate dehydrogenase complex, there was a significant change on NADH/NAD^+^ values in comparison to those found in their parental strains (Table 6). *P. putida* strains with high PHA production revealed high NADH/NAD^+^ ratio values*.* It was shown before that intracellular NADH concentrations are crucial for cell growth [41,42]. Similar observations of the importance of the NADH/NAD^+^ ratio for PHA synthesis from fatty acids in *P. putida* were published before [43-45].

## Conclusion

We successfully continued our *in silico* metabolic modeling based approach for the improvement of PHA formation in *P. putida*. The predicted overexpression of the pyruvate dehydrogenase subunit gene *acoA* in combination with the deletion of the glucose dehydrogenase gene *gcd* resulted in an increase of PHA production by 120%. Potential explanation for the observed PHA production phenotypes were derived from gene expression and cofactor quantification analyses. Beyond this work on microbial biopolymer production, *in silico* based metabolic engineering has proven truly valuable for succinate [46], 1,4 butandiol [47], bio-ethanol [48], and amino acids [49,50], suggesting this strategy as highly promising to breed superior producers industrial biotechnology.

## Methods

### Bacterial strains

*Pseudomonas putida* KT2440 (DSMZ, Braunschweig, Germany) as wild type strain and the gene knock-out mutants *P. putida* Δ*gcd*[21] were used in this study (Table 1). *E. coli* strain DH10b (Invitrogen, Darmstadt, Germany) was employed for the amplification of pJET1.2 derivatives (Promega, Darmstadt, Germany). *E. coli* XL10-Gold (Stratagene, Waldbronn, Germany) was used for replication of the pSEVA plasmids (pSEVA424, pSEVA-*pgl* and pSEVA-*acoA*). The pSEVA plasmids constitute a series of low-copy plasmids for the isopropyl 1-thio-β-D-galactopyranoside (IPTG) inducible overexpression of target genes [51]. Transformation of the *P. putida* strains was accomplished via electroporation applying a voltage of 2.5 V, 200 Ω and 25 μF, while *E.coli* DH10b was transformed as previously described [52]. *P. putida* KT2440 and its Δ*gcd* mutant were transformed with the plasmids pSEVA-*acoA* and pSEVA-*pgl* encoding the pyruvate dehydrogenase subunit A (AcoA) and 6-phosphogluconolactonase (Pgl), respectively.

### Genetic techniques and construction of bacterial strains

The 978 bp *acoA* and 729 bp *pgl* genes were PCR amplified using primer pairs *acoA*Fw/*acoA*Rv and *pgl*Fw/*pgl*Rv, respectively. Used primers sequences are listed in Table 1. The forward primers (*acoA*/Fw, *pgl*/Fw) of both genes encoded an *EcoR*I restriction site, a consensus Ribosomal Binding Site (RBS) sequence (AGGAGG), eight conserved nucleotides downstream (AAAAACAT) and 18 nucleotides from the gene of interest [51]. Both reverse primers (*acoA*/Rv, *pgl*/Rv) encoded an *Xba*I restriction site and the last 18 nucleotides of target gene sequences (Table 1). Amplified genes were cloned into pJET1.2 resulting in pJET1.2-*acoA* and pJET1.2-*pgl*, respectively. The cloned genes were excised from their host vectors via *Eco*RI and *Xba*I restriction and subsequently cloned into the *EcoR*I-*Xba*I sites of pSEVA424 resulting in pSEVA-*pgl* and pSEVA-*acoA*, respectively.

### Shake flask cultivation

*P. putida* strains were kept as frozen stock in 25% glycerol at -80°C. Prior to inoculation they were streaked on Luria Bertani agar plates and incubated for one day at 30°C. Single colonies were then picked from the plate and inoculated in 50 mL shake flasks containing 10 ml LB and incubated overnight under aerobic conditions at 30°C and shaking at 180 rpm. To start the PHA-producing process 1 L baffled shake flasks containing 200 mL of defined culture medium were inoculated to an initial OD_595_ of 0.05 and placed in a rotary shaker (180 rpm) under aerobic conditions at 30°C. Each culture was carried out by triplicate. *P. putida* strains were grown in a defined M9 mineral medium consisting of per liter 12.8 g Na_2_HPO_4_^.^7H_2_O, 3 g KH_2_O_4_, 1 g NH_4_Cl, and 0.5 g NaCl. This basic solution was autoclaved and subsequently supplemented with 0.12 g of MgSO_4_^.^H_2_O and a trace element solution made of: 6.0 mg/L FeSO_4_^.^7H_2_O, 2.7 mg/L CaCO_3_, 2.0 mg/L ZnSO_4_^.^H_2_O, 1.16 mg/L MnSO_4_^.^H_2_O, 0.37 mg/L CoSO_4_^.^7H_2_O, 0.33 mg/L CuSO_4_^.^5H_2_O, 0.08 mg/L H_3_BO_3_, and 20 g/L glucose (all filter-sterilized). Antibiotics were 62.5 μg/mL streptomycin (Sm) for *E. coli* XL10-Gold, 100 μg/mL streptomycin for *P. putida* strains and 100 μg/mL ampicillin (Ap) for *E. coli* DH10b.

### Bioreactor fermentations

*P. putida strains* were grown in M9 medium supplemented with 18.5 g/L glucose, and antibiotic as required. Bioreactor batch fermentations were carried out in a 2 L top-bench BIOSTAT B1 bioreactor (Sartorius B Systems GmbH, Melsungen, Germany) with a working volume of 1.5 L, at 30°C. The aeration rate was set to 500 mL L^-1^ min^-1^ using a mass flow controller (PR4000, MKS Instruments, Wilmington, MA, USA). The dissolved oxygen level was kept above 20% air saturation by control of the agitation speed up to a maximum of 900 rpm. The pH was maintained at 7.0 by automatic pH controlled addition of 500 mM H_2_SO_4_ or 1 M of KOH.

Cell growth was monitored via OD measurements at 600 nm (Ultraspec 2000, Hitachi, Japan). The cell dry weight was determined gravimetrically after harvesting bacteria from 10 mL culture broth for 10 min at 4°C and 10,000 rpm (Eppendorf 5810 R, Hamburg, Germany), washing with distilled water, and drying of the obtained pellet at 100°C. The ammonium concentration in cell-free extracts was measured by a photometric test (LCK 303 kit, Hach Lange, Danaher, USA). The glucose concentration in the cultivation supernatant was analyzed after appropriate dilution by HPLC chromatography (HPLC Agilent 1260, Agilent, Krefeld, Germany) through an 8 mm Rezex ROA-organic acid H column (Phenomenex, USA) at a flow rate of 0.5 mL · min^-1^ at 65°C using 0.013 N H_2_SO_4_ as the mobile phase, followed by detection using a RID detector (Agilent series1260). Gluconate and 2-keto-gluconate were quantified by HPLC chromatography using an Aminex HPX 87 H column (Biorad, Hercules, CA, USA) on a Hitachi HPLC system (Hitachi Elite LaChrom, Krefeld, Germany) and 12.5 mM H_2_SO_4_ as the mobile phase at a flow rate of 0.5 mL min^-1^ at 45°C. Compounds were quantified by UV detection at 220 nm.

### PHA characterization and quantification

Composition analyses of the polymer produced and cellular PHA content concentration determination were performed by gas chromatography (GC) and mass spectrometry (MS) of the methanolyzed polyester. For PHA production corresponding *P. putida* strains were streaked on LB agar plates and incubated for one day at 30°C. Single colonies were then picked from the plate, inoculated in a 50 mL shake flask containing 10 mL of the above described medium and incubated overnight under aerobic conditions at 30°C and 180 rpm (Innova, Enfield, USA). To begin the PHA-accumulating process, 1 L baffled shake flasks containing 200 mL of culture medium were inoculated at an initial OD_595nm_ of 0.05 and incubated under aerobic conditions at 30°C. Subsequently, 10 mL of the culture were centrifuged for 10 min at 4°C and 9,000 x *g*, followed by a washing step with distilled water and lyophilization. Methanolysis was carried out by suspending 5 – 10 mg of lyophilized cell pellet in 2 mL of chloroform and 2 mL of methanol containing 15% sulfuric acid and 0.5 mg · mL^-1^ 3-methylbenzoic acid as internal standard. Incubation followed at 100°C for 4 h. After cooling, 1 mL of demineralized water was added. The organic phase containing the resulting methyl esters of the monomers of the polymers of interest were analyzed by GC-MS on a Varian GC-MS system 450GC/240MS ion trap mass spectrometer operated by the software MS Workstation 6.9.3 (Varian Inc., Agilent Technologies, Böblingen, Germany). For this purpose, 1 mL of the organic phase was injected into the gas chromatograph at a split ratio of 1:10. Separation of the analytes of interest (i.e. the methyl esters of 3-hydroxyexanoate, 3-hydroxyoctanoate, 3-hydroxydecanoate, 3-hydroxydodecanoate, 3-hydroxy-5-cis-dodecanoate, 3-hydroxytetradecanoate) was achieved by chromatography through a FactorFour VF-5 ms capillary column (30 m X 0.25 mm i.d. X 0.25 mm film thickness). Helium was used as carrier gas at a flow rate of 0.9 mL · min^-1^. The injector and transfer line temperature were 275°C and 300°C, respectively. The temperature program was: initial temperature 40°C for 2 min, then from 40°C up to 150°C at a rate of 5°C min^-1^ and finally up to 280°C at a rate of 10°C min^-1^. Positive ions were obtained using electron ionization at 70 eV. The mass spectra were generated by scanning ions of *m/z* 50 to *m/z* 650. The PHA content (%wt) was defined as the percentage of polyhydroxyalkanoate in the cell dry weight (CDW).

### NADH and NADPH quantification

The intracellular NAD^+^, NADH, NADP^+^ and NADPH concentrations were measured employing an NADP/NADPH Colorimetric Quantitation Kit (Biovision, Abcam, UK). Crude cell lysates were obtained by undertaking various freeze-thaw lysis cycles. Cofactors concentrations were photometrically quantified by measuring colorimetric changes at 450 nm.

### Transcriptome analysis via RNA_seq_

Isolation of total RNA from corresponding 10 mL *P. putida* culture was performed using the RNeasy kit (Qiagen, Venlo, Netherland), according to the instructions provided by the manufacturer. Library preparation was done as described in [51]. Briefly, ribosomal RNA removal was performed with the MICROExpress Bacterial RNA enrichment kit using the *Pseudomonas* module (Ambion, Life Technologies, Darmstadt, Germany) according to the manufacturer’s instructions. The RNA was fragmented to a median fragment size of 200 nt by sonication using Covaris Adaptive Focused Acoustics device (Covaris, LGC Genomics, Herts, UKStrand-specific cDNA libraries were generated by previous RNA-Adapter ligation and subsequent reverse transcription. Hereby the ligated 5′-RNA-Adapter contained one of 24 different 6 nt barcode sequences [51], which allowed multiplexing of several libraries in a single Illumina lane for sequencing. Up to 20 libraries were pooled and treated with duplex-specific nuclease (DSN) for additional rRNA removal. Sequencing was performed on an Illumina HiSeq generating single-end reads with a length of 50 base pairs. Computational analysis of the raw sequence data obtained in Illumina FASTQ-format was performed as described previously by [53], with some modifications Mapping was performed using *stampy,* a short-read aligner that allows for gapped alignments [54] for quantification of gene expression. The reads per gene (rpg) values of all genes were calculated from the SAM output files. Testing for differential expression against KT2440 (four biological replicates) was performed with DESeq [55], an R software package that uses a statistical model based on the negative binomial distribution.

### Protein quantification

After 60 hours, 10 mL cultures were withdrawn and cells were harvested by centrifugation. Proteins from up to 50 μg cell pellet were separated via SDS-PAGE [56]. Resulting SDS gels were stained using Coomassie Brillant Blue and protein amounts quantified densiometrically on a GS-800 scanner device.

## Competing interests

The authors declare that they have no competing interests.

## Authors’ contributions

IPC designed the study and composed the manuscript. JB and IPC have made substantial contributions to the acquisition and analysis of data. AB and SH have made contribution of acquisition of the transcriptome data. MS and CW have been involved in the analysis of the data. MJ, JB, IPC, and DJ have been involved in writing the manuscript critically for important intellectual content. All authors read and approved the final version of the manuscript.
